# Delegated multi-party private set intersections from extendable output functions

**DOI:** 10.7717/peerj-cs.3141

**Published:** 2025-08-29

**Authors:** Aslı Bay

**Affiliations:** Department of Computer Engineering, Faculty of Engineering and Natural Sciences, Antalya Bilim University, Döşemealtı, Antalya, Turkey

**Keywords:** Extendable output functions, Multi-party computation, Private set intersections

## Abstract

Operations on sensitive datasets from different parties are essential for various practical applications, such as verifying shopping lists or enforcing no-fly lists. Traditional methods often require one party to access both datasets, which poses privacy concerns. Private set operations provide a solution by enabling these functions without revealing the data involved. However, protocols involving three or more parties are generally much slower than unsecured methods. Outsourced private set operations, where computations are delegated to a non-colluding server, can significantly improve performance, though current protocols have not fully leveraged this assumption. We propose a new protocol that removes the need for public-key cryptography. Our non-interactive set intersection protocol relies solely on the security of an extendable output function, achieving high efficiency. Even in a ten-client setting with 16,384-element sets, the intersection can be computed in under 54 s without communication overhead. Our results indicate that substantial performance improvements can be made without sacrificing privacy, presenting a practical and efficient approach to private set operations.

## Introduction

Many aspects of our daily lives revolve around set operations, but in some cases, these operations involve sets held by different parties, and the data in them is sensitive. For example, finding suitable meeting data across multiple private calendars involves a set intersection between multiple parties, which must not reveal any information besides when all parties are available. This problem asks for a multi-party private set intersection (MPSI), where parties ${\scr {P}}_1, \ldots ,{\scr {P}}_n$ each hold a private set ${X_{1}}, \ldots ,{X_{n}}$, and collectively compute ${X_{1}} \cap \ldots \cap {X_{n}}$.

MPSI protocols have a variety of use cases. For example, Bay et al. (2022) mention online recommendation systems, including dating sites; confidential data sharing, such as security incident information; border protection against criminal attempts; and network security operations, such as botnet detection and detecting intrusions by finding the sets’ suspicious internet protocols (IPs) within the sets. Moreover, they are a valuable tool for solving problems in cyber threat intelligence (Vos, Erkin & Doerr, 2021).

This article considers a special setting to compute the functionality of an MPSI, where an outsourcing server ${\scr {P}}_{{{\mathrm{srv}}}}$ exists that does not collude with any of the other parties, which we shall now refer to as clients. One of the clients receives the result of the operations. We refer to this client, denoted as ${\scr {P}}_q$, as the querying client or the querier. Previous work has shown that this setting leads to significant performance improvements (Abadi et al., 2022).

In this article, we investigate the existence of a protocol for outsourced MPSIs that relies solely on extendable output functions (XOFs), a specific type of hash function. XOFs are among the most efficient cryptographic primitives, typically relying on weaker security assumptions. Indeed, we propose such a protocol: which is non-intereactive, but it does leak information to the server about the size of the intersection and the size of the querying client’s set. We prove that this protocol is secure in this security model using a simulation-based proof in the random oracle model, assuming that the communication channels with the server are private.

At the same time, we raise questions about the validity of this security setting, which we refer to as the *non-colluding server setting*. Not only is it hard to realize this setting in practice, but the security of protocols in this setting relies entirely on secret information known to all clients never leaking to the server, or the protocol instantly breaks. This is a fundamental problem of the setting that our protocol highlights. We show that our protocol and previous work entirely rely on the “free unlinkability” provided by a keyed hash function or permutation for which the key is unknown to the server.

By showing that this setting allows outsourced MPSI to be performed non-interactively by relying only on XOFs, we argue that many previous works rely on excessively strong security assumptions and computationally heavy cryptographic primitives (Abadi, Terzis & Dong, 2015; Kerschbaum, 2012a; Wang et al., 2021). An exception comes from Feather (Abadi et al., 2022), among others, which only relies on other symmetric primitives such as permutations. One downside is that Feather leaks the access pattern of the elements queried. On the other hand, Feather allows the parties to update the elements in the server and forget about their own sets.

The protocol we propose relies on secret shares generated non-interactively using multiple calls to an XOF. By generating shares in this way, we realize collusion resistance between up to $n - 2$ clients. These clients encode their sets as Bloom filters and use the generated secret shares to compute an AND operation between their respective Bloom filters to retrieve a Bloom filter encoding the intersection. This approach is similar to previous works on MPSI (Bay et al., 2022; Vos, Conti & Erkin, 2022; Debnath et al., 2021). One key difference is that the Bloom filter now must rely on a cryptographically secure hash function rather than a statistical hash function. The reason is that the server may not learn the relation between hashes and elements. We use the same XOF to instantiate this cryptographic hash function. To eliminate privacy leakage, we configure our Bloom filters with a single hash function ( $h = 1$), which prevents the server from inferring which elements are likely present in the input sets. Despite this restriction, our protocol achieves high efficiency due to the performance of XOFs. Experimental results show that an intersection involving ten clients, each holding more than 16,000 elements, can be computed in only 54 s, excluding communication delays.

To summarize, our contributions are as follows:

- We introduce a novel outsourced MPSI protocol that relies solely on XOFs, eliminating the need for public-key cryptographic primitives. We provide a formal security proof of our protocol in the semi-honest model using a simulation-based approach in the random oracle model.
- We perform a comprehensive performance evaluation of our protocol, including comparisons with Feather (Abadi et al., 2022), a recent outsourced private set intersection (PSI) protocol based on symmetric keys. Our results show that our protocol offers improved scalability and competitive performance, especially as the number of clients increases.
- We benchmark our protocol with multiple XOFs, including BLAKE3-XOF, SHAKE128-XOF, and SHAKE256-XOF. The results demonstrate that BLAKE3-XOF offers significant efficiency advantages, especially in larger-scale scenarios.
- We analyze the effects of network constraints (latency and bandwidth) and show that the protocol remains practical even under limited communication conditions.
- We draw attention to critical limitations in the non-colluding server model and discuss its practical challenges, including the consequences of partial collusion.
- We open-source a proof-of-concept implementation of the protocol written in C++.^1^ 1The implementation can be found at: https://doi.org/10.5281/zenodo.15469698

In the remainder of this article, we review relevant prior work in “Related Work”. “Preliminaries” provides background on extendable output functions (XOFs) and Bloom filters. “Protocol for Intersections Between All Clients” introduces our proposed MPSI protocol, explaining its design, setup, and operational steps. “Proof of Security” presents a formal security analysis under the semi-honest model and explores the limitations of the non-colluding server assumption, including potential collusion risks. “Results” evaluates the protocol’s performance through extensive experiments. Finally, in “Conclusion”, we summarize our contributions and discuss directions for future research.

## Related work

### Traditional private set intersection protocols

Traditional private set intersection (PSI) protocols enable clients to compute the intersection of their datasets while keeping their private data local and secure. These protocols typically involve direct client-to-client or client-to-server interaction, assuming all parties have sufficient computational resources. Since the introduction of PSI by Freedman, Nissim & Pinkas (2004), it has been a key focus in privacy-preserving computations. Their protocol utilizes homomorphic encryption and balanced hashing to compute set intersections securely.

Following this foundational work, various extensions have been developed with enhanced security models and functionalities. For instance, Kissner & Song (2005) implement multiparty intersection computation using the polynomial representation of sets combined with additive homomorphic encryption. Building on these advancements, (Hazay & Lindell, 2008) leverage oblivious pseudorandom functions in their PSI construction. To enhance security against malicious adversaries, Dachman-Soled et al. (2009) develop a robust PSI protocol using homomorphic encryption, enhanced Shamir secret sharing, cut-and-choose techniques, and zero-knowledge proofs. In a similar direction, Hazay & Nissim (2010) later develop a PSI protocol specifically designed for the malicious adversarial model, based on Freedman’s earlier work. Moreover, Camenisch & Zaverucha (2009) introduce a PSI protocol requiring signed input sets, which need verification from a trusted certifying authority. De Cristofaro, Kim & Tsudik (2010) also devise an efficient PSI protocol for malicious settings, employing public-key encryption and hash functions to improve performance. Ateniese, De Cristofaro & Tsudik (2011) design a protocol that conceals the size of the clients’ input set for added privacy.

In the semi-honest model, Huang, Evans & Katz (2012) propose a garbled circuit-based PSI protocol, achieving $O(n\log n)$ complexity for symmetric key operations, where $n$ represents the set size. Meanwhile, Many, Burkhart & Dimitropoulos (2012) introduce a PSI protocol using a Bloom filter, where parties query the filter to obtain the intersection. However, this method leaks information about the other party’s set, making it insecure.

Furthermore, Dong, Chen & Wen (2013) design two different PSI protocols: one for semi-honest adversaries and another for malicious adversaries, both relying on the garbled Bloom filter intersection and oblivious transfer, making them scalable for large dataset processing. Later the same year, Dong et al. (2013) propose a fair mutual PSI protocol relying on a trusted third party, allowing both parties to receive the output. However, this approach incurs high costs due to the dependence on zero-knowledge proofs and oblivious polynomial evaluation.

This line of work is further refined by Debnath & Dutta (2015), making Bloom filter-based PSI a viable solution for large-scale private set operations. Similarly, Bay et al. (2022), Vos et al. (2024) have extended these improvements with further optimizations based on Bloom filters. Moreover, Cuckoo filters (Pinkas et al., 2018; Jiang et al., 2023) have been widely adopted to reduce memory and computation costs.

In parallel, Pinkas, Schneider & Zohner (2014) propose PSI protocols utilizing IKNP-OT (Ishai et al., 2003), achieving notable improvements in computational efficiency at the cost of increased communication. Further efficiency gains are achieved by Kolesnikov et al. (2016), who leverage Oblivious Transfer Extension (OTE) to construct a batched oblivious pseudorandom function. Their method achieves two- to three-fold improvement in computational efficiency for two-party PSI, making it one of the fastest protocols in high-speed networks. Following these advancements, Rindal & Rosulek (2017) improve PSI efficiency by utilizing hashing schemes to reduce complexity and by employing the dual-execution technique from Mohassel & Franklin (2006). Subsequently, Kolesnikov et al. (2017) pioneer the oblivious programmable pseudorandom function technique, offering an efficient solution with symmetric-key operations and laying the foundation for later works like secret-shared PSI by Rindal & Schoppmann (2021).

Furthermore, bitset-based methods combined with homomorphic encryption (Ruan et al., 2019; Bay et al., 2021) have been proposed. Key agreement protocols such as those in Rosulek & Trieu (2021), Wei et al. (2024) have also facilitated secure PSI computations between multiple parties. Finally, the emergence of quantum computing has prompted researchers to explore quantum PSI protocols, which promise enhanced security against quantum attacks while reducing communication complexity (Shi et al., 2015; Liu, Zhang & Shi, 2020).

Overall, traditional PSI protocols rely on direct interaction between parties and do not support data outsourcing. Hence, each party needs to store its data locally, leading to significant storage overhead. Furthermore, these protocols require continuous online participation from all parties throughout the computation, increasing communication costs and limiting scalability, particularly in distributed or resource-constrained environments.

### Outsourced PSI protocols

Outsourced/delegated private set intersection (OPSI) protocols address scenarios where clients have limited computational resources by introducing a third-party, typically a cloud server, to perform the intersection computation. In this model, clients delegate the computationally intensive set intersection task to the server, thus benefiting from its storage and processing capabilities.

In this section, we provide a structured overview of existing OPSI protocols, categorizing them based on their cryptographic foundations into *symmetric-based* and *asymmetric-based* protocols, highlighting their contributions and limitations. A detailed comparison is presented in Table 1.

**Table 1:** The comparison of delegated PSI protocols.

	Functionality			Computation		Communication		Security		
	$n > 2$	Rep.	Upd.	Compl.	Only symmetric	Compl.	Non-interactive	Size-hiding	Mal.	Hardness
Abadi et al. (2022)	$\bullet$	$\bullet$	$\bullet$	$O(k)$	$\bullet$	$O(k)$	$\bullet$	$\bullet$	$\circ$	PRF
Abadi, Terzis & Dong (2015)	$\bullet$	$\bullet$	$\circ$	$O(k)$	$\circ$	$O(k)$	$\bullet$	$\bullet$	$\circ$	DCR
Kerschbaum (2012a)	$\bullet$	$\circ$	$\circ$	$O({k^{2}})$	$\circ$	$O({k^{2}})$	$\circ$	$\bullet$	$\circ$	RSA
Wang et al. (2021)	$\circ$	$\bullet$	$\circ$	$O(k)$	$\circ$	$O(k)$	$\bullet$	$\bullet$	$\circ$	DBDH
Abadi, Terzis & Dong (2016)	$\bullet$	$\bullet$	$\circ$	$O(k)$	$\circ$	$O(k)$	$\bullet$	$\bullet$	$\bullet$	AHE
Abadi et al. (2019)	$\bullet$	$\bullet$	$\circ$	$O(k)$	$\bullet$	$O(k)$	$\bullet$	$\bullet$	$\circ$	PRF
Kamara et al. (2014)	$\bullet$	$\circ$	$\circ$	$O(k)$	$\bullet$	$O(k)$	$\circ$	$\bullet$	$\bullet$	PRP
Kerschbaum (2012b)	$\circ$	$\circ$	$\circ$	$O({k^{2}})$	$\circ$	$O({k^{2}})$	$\bullet$	$\bullet$	$\circ$	QR
Kumar et al. (2021)	$\circ$	$\bullet$	$\circ$	$O(k)$	$\circ$	$O(k)$	$\bullet$	$\bullet$	$\bullet$	DDH
Liu et al. (2014)	$\bullet$	$\circ$	$\circ$	$O(k)$	$\bullet$	$O({k^{2}})$	$\bullet$	$\circ$	$\circ$	–
Wang et al. (2022)	$\circ$	$\bullet$	$\bullet$	$O(k)$	$\circ$	$O(k)$	$\bullet$	$\circ$	$\bullet$	q-SBDH
Zhang et al. (2017)	$\bullet$	$\circ$	$\circ$	$O(k)$	$\bullet$	$O(k)$	$\bullet$	$\circ$	$\bullet$	LWE
Zheng & Xu (2014)	$\bullet$	$\circ$	$\circ$	$O(z)$	$\circ$	$O(k)$	$\bullet$	$\circ$	$\bullet$	DL
Yang et al. (2018)	$\bullet$	$\bullet$	$\bullet$	$O({k^{2}})$	$\circ$	$O(k)$	$\bullet$	$\bullet$	$\circ$	RSA
Sun et al. (2023)	$\bullet$	$\bullet$	$\bullet$	$O(k)$	$\circ$	$O(k)$	$\bullet$	$\bullet$	$\circ$	GDDHH
Jiang et al. (2024)	$\circ$	$\circ$	$\circ$	$O(klogk)$	$\bullet$	$O(k)$	$\bullet$	$\circ$	$\circ$	PRF
Ours	$\bullet$	$\circ$	$\bullet$	$O(k)$	$\bullet$	$O(k)$	$\bullet$	$\circ$	$\circ$	XOF

**Note:**

n: number of parties, k: set size, z: intersection size, Rep: Repeated, Upd: Updatable, Mal: Malicious, DCR: Discrete Composite Residuosity, AHE: Additively Homomorphic Encryption, QR: Quadratic Residuosity, PRF: Pseudo-Random Function, DDH: Decional Diffie-Hellman, q-SBDH: Bilinear q-strong Diffie-Hellman, DBDH: Decisional Bilinear Diffie-Hellman, GDDHH: General Decision Diffie-Hellman Exponent, LWE: Learning with Error, DL: Discrete Logarithm, XOF: Extendable Output Function

#### Asymmetric-based OPSI protocols

Asymmetric cryptographic tools are widely used in OPSI protocols that rely on public-key primitives such as Rivest–Shamir–Adleman (RSA), Diffie-Hellman, and bilinear pairings. These primitives provide strong security guarantees at the cost of introducing computational overhead.

A well-known example of an outsourced PSI protocol is the one proposed by Kerschbaum (2012a), which utilizes RSA encryption. In this protocol, a client encrypts and outsources their dataset to the server, which then computes the intersection on behalf of the client. However, a key limitation of this approach is that each intersection computation requires re-encrypting and re-uploading the dataset, making it inefficient for repeated use scenarios. To enhance efficiency, Kerschbaum (2012b) propose a protocol leveraging Bloom filters and additively homomorphic encryption (AHE). In this scheme, clients encrypt their datasets using Bloom filters before sending them to the cloud server, which then computes the intersection. This approach still requires fresh computations for each intersection, although it reduces the overall computational overhead compared to the prior method.

Another significant advancement is O-PSI (Abadi, Terzis & Dong, 2015), which represents datasets as point-value polynomials and employs AHE to maintain privacy. However, this scheme has been shown to be vulnerable to man-in-the-middle attacks and collusion threats, which were later mitigated in Oliaee et al. (2018). Building upon this, the verifiable delegated private set intersection (VD-PSI) in Abadi, Terzis & Dong (2016) provides verifiability using an AHE scheme called Paillier PKI, allowing clients to verify correctness without retaining a local copy. In parallel, the work by Zheng & Xu (2014) utilizes bilinear mappings and a bilinear map accumulator to verify computational accuracy, thus contributing to verifiable outsourced PSI research. Yang et al. (2018) propose a PSI protocol based on the RSA cryptosystem, similar to Abadi, Terzis & Dong (2015), but eliminate the need for clients to encode their datasets before outsourcing jointly.

More recent protocols, such as tag-based verifiable delegated private set intersection (TVD-PSI) (Wang et al., 2022), extend this approach by integrating tag-based classification. However, this introduces additional computational workload due to the need to manage tags and handle dynamic data updates. Revocable and verifiable private set intersection (RV-PSI) (Sun et al., 2023) leverages the General Decision Diffie-Hellman Exponent problem for non-interactive revocable PSI computation. Its reliance on complex cryptographic primitives makes it resource-heavy, especially for revocation and verification tasks. Additionally, the protocol exposes the exact number of elements in one of the intersecting sets, creating a privacy leakage.

More recent protocols such as Kumar et al. (2021) and Wang et al. (2021) further enhance security by relying on Diffie-Hellman and Decisional Bilinear Diffie-Hellman (DBDH) assumptions. These solutions are particularly well-suited for malicious security settings and offer different authorization levels to cloud servers. Despite these advancements, the main drawback of asymmetric-based protocols is their computational complexity, making them less efficient for large-scale PSI computations.

#### Symmetric-based outsourced PSI protocols

To mitigate the inefficiencies of asymmetric-based protocols, researchers have proposed symmetric-based approaches that use hash functions, pseudorandom permutations, and symmetric encryption techniques.

An early notable development is the work in Liu et al. (2014), which applies hash functions and symmetric encryption to delegated PSI. This approach allows repeated computations but has privacy issues due to vulnerabilities in its set encoding method. Specifically, by separately performing PSI for multiple clients, the server may infer intersections without consent. Later, Kamara et al. (2014) explore outsourced PSI on large datasets using pseudorandom permutations. Despite scalability, communication and memory usage can become substantial for very large datasets.

In a subsequent advancement, Abadi et al. (2019) introduce a relatively new delegated PSI protocol named EO-PSI. Unlike O-PSI (Abadi, Terzis & Dong, 2015), their protocol eliminates AHE and relies on hash tables and point-value polynomial representations, while still supporting repeated delegation. Nevertheless, security weaknesses were identified in Kavousi, Mohajeri & Salmasizadeh (2020), leading to an improved version that removes reliance on secure channels.

In a separate line of work, Zhang et al. (2017) propose a protocol based on the hardness of the Learning with Errors problem, incorporating a reputation system to penalize protocol violations. While this approach improves security, it introduces high communication overhead between clients. Moreover, the protocol relies on a strong security assumption, and its guarantees collapse if collusion occurs between a server and a client or between two servers.

A recently published protocol named Feather (Abadi et al., 2022) introduces an efficient symmetric-based MPSI approach, avoiding public-key tools entirely. The protocol is built upon three main components: a hash table, polynomial-based encodings, and Bloom filters—enabling efficient private set intersection and dynamic updates. However, one limitation is its access pattern leakage. Despite this, Feather allows clients to update elements on the server without maintaining local copies. Additionally, Jiang et al. (2024) introduce verifiable outsourced private set Intersection (VO-PSI) protocol that ensures correctness even when cloud servers behave maliciously.

#### Motivation for our work

To summarize, many outsourced MPSI protocols depend on strong security assumptions and costly cryptographic operations. Others, like Feather, improve efficiency by using symmetric cryptography, though they still face limitations in privacy and scalability. Our work addresses these limitations by introducing a novel protocol that exclusively leverages extendable output functions (XOFs), ensuring non-interactive secret sharing, efficient computation, and resistance to collusion among up to $n - 2$ clients, while preventing server inference through Bloom filters and secure hashing. Compared to Feather, our protocol differs in several key aspects mentioned below:

- *Privacy:* Feather leaks access patterns—revealing which data locations (*e.g*., memory cells, Bloom filter bits) are accessed or modified. This can enable a server to infer sensitive information. Our protocol avoids this by not leaking access patterns, though it does reveal the intersection size and the querying client’s set size.
- *Efficiency:* Feather works well in small two-party setups, whereas our protocol scales more effectively. For instance, ours takes 54 s to finish with 10 clients and 16,384-element sets, while Feather takes 62.76 s. Our effective XOF-based masking causes this gap to widen with more clients or larger datasets.
- *Security Model:* Feather uses permutations and other symmetric tools. Relying solely on XOFs, our protocol is simpler and removes extra cryptographic dependencies. It also supports non-interactive secret sharing and addresses core privacy challenges directly in the non-colluding server setting.

## Preliminaries

In this section, we briefly explain how XOFs are used to generate secret shares non-interactively and how Bloom filters are used to compute set intersections. The notation we use can be found in Table 2. Note that all variables are zero-indexed except for the bins in a Bloom filter, so we have that ${\scr {H}}_j(x) \in \{ 0, \ldots ,m\}$.

**Table 2:** Summary of the symbols used in this work.

Symbol	Definition
$n$	Number of clients
${\scr {P}}_i$	Client number $i$
${\scr {P}}_q$	The querying client
${\scr {P}}_{{{\mathrm{srv}}}}$	The server
${X_{i}}$	Client ${\scr {P}}_i$’s private set
$\scr {U}$	The universe of possible set elements
$k$	Upper bound for a client’s set size
$m$	Number of bins in the Bloom filter
$h$	Number of hash functions in the Bloom filter
${\scr {H}_j}(x)$	Hash of element $x$ with seed $j$, in $\{ 0, \ldots ,m - 1\}$
$A[\_]$	Indexes vector *A*
$B{[\_]_{40}}$	Indexes bitstring *B* by 40-bit segments

### XOR-sharing using XOFs

An XOF takes an input message and an output size $d$, and produces a cryptographically secure digest of $d$ bits. In our delegated PSI protocol, large masks are needed to ensure that only the intersection of sets is revealed. Consequently, XOFs are effective for this purpose because they can generate these large masks from secret values, and the masks have the property that their XOR combination results in zero. Specifically, if each pair of clients $({\scr {P}}_i,{\scr {P}}_{i^\prime })$ share a secret value ${s_{i,i^\prime }} = {s_{i^\prime ,i}}$ that only they know, where $i,i^\prime \in \{1, \ldots ,n\}$, we can use XOFs to generate large masks *M* that combine to zero with high performance, relying on the self-inverse property of the XOR function:

(1) $${M_{i}} \leftarrow \mathop \oplus \limits_{i \ne i^\prime }{ \rm XOF}_{d}({s_{i,i^\prime }}).$$

Here, $\oplus$ denotes the bitwise XOR operation applied across all relevant terms, and ${\rm XOF}_{d}({s_{i,i^\prime }})$ represents the output of the XOF applied to the secret ${s_{i,i^\prime }}$, producing a digest of $d$ bits.

This technique is essentially a non-interactive version of the dining cryptographer’s problem (Chaum, 1988), in which each party’s randomness is replaced by a keyed pseudo-random function in the form of an XOF.

### Bloom filters for set intersections

Many previous works perform set intersections privately by first representing the sets as Bloom filters (Bay et al., 2022; Debnath et al., 2021; Miyaji, Nakasho & Nishida, 2017). A bloom filter is a data structure with $m$ bins that are either $0$ or $1$. It is indexed by $h$ hash functions, so that when queried, the result is only $1$ when all indexed bins are indeed $1$. This corresponds to an AND operation. To create a Bloom filter representing a set *X*, one iterates over all elements $x \in X$ and evaluates hashes ${\scr {H}}_j(x) \in \{ 0, \ldots ,m-1\}$ for $j = 1, \ldots ,h$. The bins indicated by the hash functions are set to $1$.

To compute the Bloom filter representing the intersection, we only have to perform an AND operation between the respective bins of the input Bloom filters. To find the intersection from its Bloom filter representation, one must perform a query for each element in one of the original sets. Suppose the intermediate Bloom filter is leaked to any of the parties. In that case, they can learn the distribution of the elements contained in the original input sets when the number of hash functions $h > 1$ (Vos, Conti & Erkin, 2022). We use cryptographic hash functions in this work to obfuscate the resulting Bloom filter from the server so that the server does not know the relationship between the $1$s in the Bloom filter and the elements in the universe $\scr {U}$. This does reveal information about the cardinality of the intersection to the server.

Membership queries in Bloom filters are approximate because they might return false positives. We denote the probability of false positives by $\varepsilon$, which is the probability that a query gives the wrong result. Goel & Gupta (2010) provide an upper bound for $\varepsilon$ when *N* elements have been inserted in a Bloom filter:

(2) $$\varepsilon \le {\left( {1 - {e^{ - {{h(N + 0.5)} \over {m - 1}}}}} \right)^h}.$$

In practice, we only tolerate a maximum probability of false positives $\varepsilon$. So, we want to select the most compact Bloom filter to satisfy this constraint, which leads to a convex minimization problem (Vos, Conti & Erkin, 2022):

(3) $${\min _{h \ge 1}} \left \lceil - {{h(N + 0.5)} \over {\ln 1-\root h \of \varepsilon }} \right \rceil + 1 .$$

## Protocol for intersections between all clients

We present a protocol for outsourced private set intersections between all clients, which only relies on XOFs. This protocol only requires the clients to communicate with the server once and the result is given to the querying client ${\scr {P}}_q$. This protocol obtains the intersection by first representing sets as Bloom filters. The key insight is that we can efficiently compute the AND operation between these Bloom filters using XOR-secret sharing because the server is non-colluding. As a result, the server only learns information about cardinalities when $h = 1$ rather than the actual elements contained in the intersection. When $h > 1$, some information about the distribution of elements is leaked. We provide and analyze the protocol for the general case of $h$ but for a fair comparison with related work, $h = 1$. The clients do not learn anything beyond their input, apart from the querying client, who receives the final output.

### Setup of the protocol

Our protocol relies on private channels between each client and the server, which must be set up ahead of time. Next, we rely on the fact that there exists a shared random seed between each pair of clients $({\scr {P}}_i,{\scr {P}}_{i^\prime })$, which must stay secret. While it is possible to let a third party generate these seeds, the parties can also generate them collectively using a series of pairwise Diffie-Hellman key exchanges. As a consequence, the network topology would go from a star to a full mesh. In the setup, the clients must also agree on a set of hash functions ${\scr {H}}_j$ for $j = 1, \ldots ,h$, without the server learning them. One possibility is for the clients to engage in a random coin toss together and use the resulting randomness as a key for the cryptographic hash functions. Finally, all clients must agree on the size of the Bloom filter $m$, which is not secret.

### Computing the intersection

**Figure 1:**
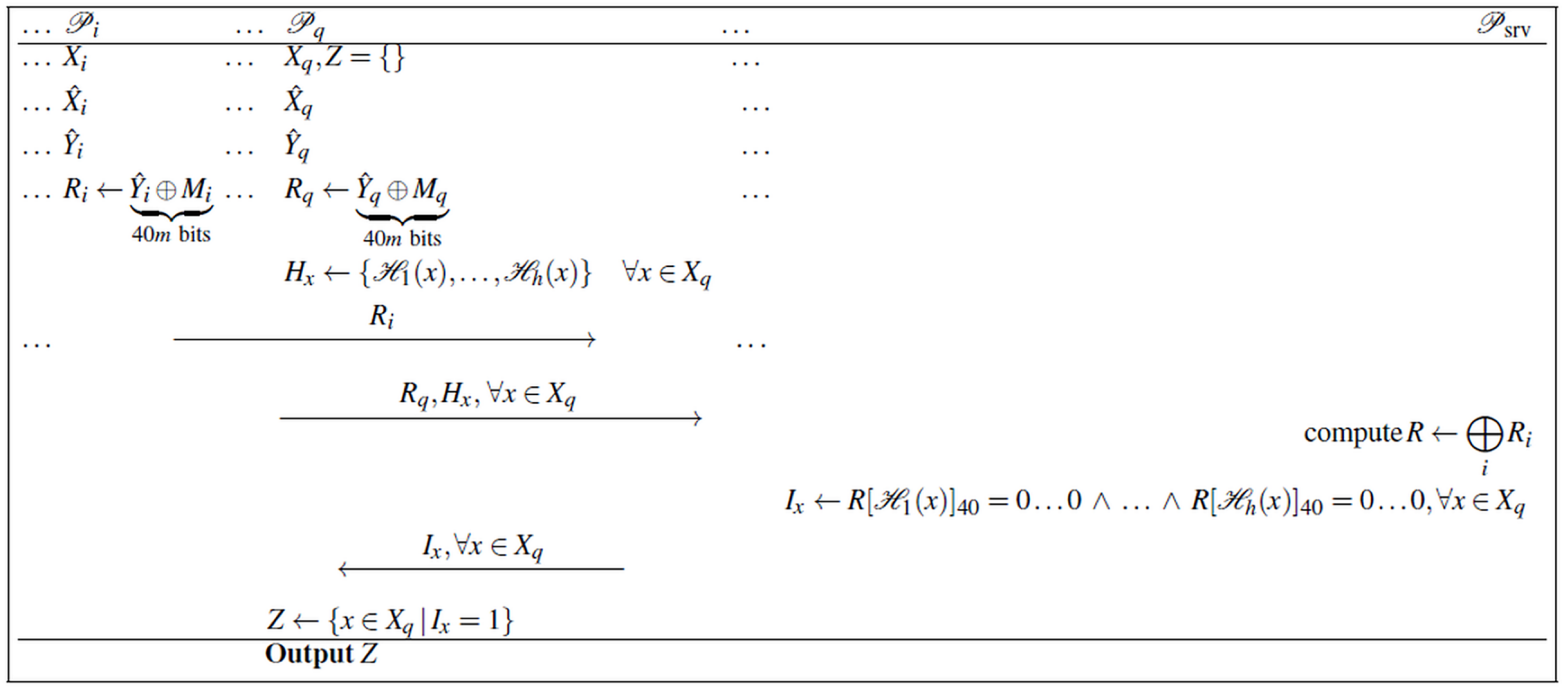
Efficient delegated MPSI protocol using only symmetric primitives, while only leaking information about $|{X_{1}} \cap \ldots \cap {X_{n}}|$ and $|{X_{q}}|$ to the server.

Each client ${\scr {P}}_i$, $1 \le i \le n$ has input set ${X_{i}}$ and collective hash functions ${\scr {H}}_1, \ldots ,{\scr {H}}_h$, which are unknown to the server. The server ${\scr {P}}_{{{\mathrm{srv}}}}$ has no inputs. Client ${\scr {P}}_q$ is the querying client, who receives the final intersection. See Fig. 1 for the depiction of the protocol; the steps of the protocol are enumerated as follows:

1. Each client ${\scr {P}}_i$ encodes their set ${X_{i}}$ as a Bloom filter ${\hat X_{i}}$.
2. Each client ${\scr {P}}_i$ transforms all zeroes in ${\hat X_{i}}$ to 40 bits of randomness and all ones to zeroes:
${\hat Y_{i}}{[t]_{40}} \leftarrow \left\{ {\matrix{ {{r_{i,x}}} \hfill & {{\mathrm{if}}\, {{\hat X}_i}[t] = 0} \hfill \cr  {0 \ldots 0} \hfill & {{\mathrm{if\;}} {{\hat X}_i}[t] = 1} \hfill \cr  } } \right.$
where ${r_{i,x}} \in {\{ 0,1\} ^{40}}$ and $t = 0, \ldots ,m - 1$.
3. Each client ${\scr {P}_i}$ computes ${M_{i}}$ using Eq. (1), then computes the message:
${R_{i}} \leftarrow \underbrace {{{\hat Y}_i} \oplus {M_{i}}}_{\rm40\,m{\mathrm{\;bits}} }$
and sends ${R_{i}}$ to the server ${\scr {P}_{{{\mathrm{srv}}}}}$.
4. The querying client ${\scr {P}}_q$ computes:
$H \leftarrow \{ {\scr {H}}_1 (x), \ldots ,{\scr {H}}_h (x)\} \quad \forall x \in {X_{q}}$
and sends it to the server ${\scr {P}}_{{{\mathrm{srv}}}}$.
5. The server ${\scr {P}}_{{{\mathrm{srv}}}}$ aggregates all ${R_{i}}$ into $R \leftarrow \mathop \oplus \nolimits_{i} {R_{i}}$. We refer to this XOR-based combination of masked Bloom filters as *aggregated masking*.
6. The server checks whether the 40-bit segments of *R* indicated by the querying client are all zero:
${I_{x}} \leftarrow R{[{\scr {H}}_1(x)]_{40}} = 0 \ldots 0\; \wedge \; \ldots \; \wedge \;R{[{\scr {H}}_h(x)]_{40}} = 0 \ldots 0$
for all $x \in {X_{q}}$, and sends the result to the querying client ${\scr {P}}_q$.
7. The querying client ${\scr {P}}_q$ **outputs** $Z \leftarrow \{ x \in {X_{q}}\mid {I_{x}} = 1\}$.

Notice that the output of XOFs plays a crucial role in concealing the 40-bit zeros present in ${\hat Y_{i}}[j]$, which indicate the presence of an element in the set. These 40-bit zeros will effectively disappear when the messages ${R_{i}}$’s from all parties are combined, as ${M_{1}} \oplus {M_{2}} \oplus \ldots \oplus {M_{n}} = 0$. In the protocol description, we chose to work over 40-bit secret shares. While it is possible to choose smaller shares, this would result in a higher probability of a $1$ in the Bloom filter accidentally turning into a $0$. Such a phenomenon would lead to false negatives, which makes studying the protocol’s correctness significantly harder. By choosing 40 bits, the probability of this occurring is negligible at ${2^{ - 40}}$.

This protocol is correct because the server ${\scr {P}}_{{{\mathrm{srv}}}}$ only returns the index of $x$, a data set element of ${\scr {P}}_q$, when $R{[{\scr {H}}_j(x)]_{40}} = 0$ for all $j \in \{ 1, \ldots ,h\}$, which reflects a Bloom filter query. This happens only when $x$ appears in every ${X_{i}}$ that is $x \in {X_{1}} \cap \ldots \cap {X_{n}}$. Due to how we use Bloom filters in our protocol, $1$ s are replaced with 40 $0$ s, and $0$ s are replaced with 40-bit random bitstrings. This requires the value coming from all Bloom Filters to be 0 in that index for an element to exist in the intersected set. If even one is non-zero, a random number appears where that index is, thereby hiding the elements that are not in the intersection.

### Updating the private sets

In Feather (Abadi et al., 2022), clients can update their inputs held by the server. We can use a similar trick to update inputs if the server has not yet revealed the intersection. For one client to update their private set, they must be able to turn a $0$ in the set representation to a $1$ or a $1$ to a $0$. They can do so for bin $j$ by knowing ${r_{i,j}}$. The client simply tells the server which segment $j$ to update and sends along ${r_{i,j}}$. The server XORs this value into the corresponding segment, flipping the encoded bit. This does reveal the access pattern to the server, as is also the case for Feather.

### Efficiency

The computational effort for a client is dominated by the computation of the extendable output functions. The asymptotic run time of XOFs scales linearly with the number of bits in the output, which is $\rm 40\, m$ in our case. As shown in “Concrete Communication Cost Analysis” of Vos, Conti & Erkin (2022), $m = O(k)$. Since each client must execute $n - 1$ XOFs, the total asymptotic complexity is $O(nk)$. Communication-wise, each client only sends one message, which is exactly $\rm 40\,m$ bits. So, in the same way, the asymptotic complexity is $O(k)$. The querying client sends another $hk$ elements of at most ${\log _{2}}(m)$ bits, so its complexity is $O(hk\log m)$.

In theory, the server only has to aggregate $h$ bins of the masked Bloom filters it received for each element. In other words, in the worst case, the server must perform $O(nkh)$ XOR operations. When it comes to communication, the server receives $n$ messages of length $\rm 40\,m$, but it only sends one message back to the querying client, which has length $hk$ in the worst-case. So, asymptotically, the server sends $O(hk)$ bits.

## Proof of security

In this section, we prove our protocol to be secure in the semi-honest model for a non-colluding server with private channels. Here, we do not consider updating the elements held by the server. We give a simulation-based proof in the random oracle model. In other words, we replace the XOF and hash functions with random oracles that always output true randomness unless they are queried for the same element again.

Before we proceed, we prove the following lemma about Eq. (1).

**Lemma 1.** *Given ${M_{1}}, \ldots ,{M_{n}}$ from Eq. 1, $M{^\prime _{2}}, \ldots ,M{^\prime _{n}}{ \in _{{\mathrm{R}}}}{\{ 0,1\} ^{\rm 40\,m}}$, and $M{^\prime _{1}} \leftarrow M{^\prime _{2}} \oplus \ldots \oplus M{^\prime _{n}}$, then it holds that $\{ {M_{1}}, \ldots ,{M_{n}}\} \mathop \equiv \limits^{{\mathrm{s}}} \{ M{^\prime _{1}}, \ldots ,M{^\prime _{n}}\}$ in the random oracle model*.

Proof. We recall that:

${M_i \gets \oplus_{i \ne i'} {\rm xof}_d(s_{i,i'})}$

so each share ${M_{2}}, \ldots ,{M_{n}}$ contains one term that does not exist in any of the other shares, namely ${\rm XOF}_{d}({s_{2,1}}), \ldots ,{\rm XOF}_{d}({s_{n,1}})$, respectively. Since we model the XOF as a random oracle, these terms are statistically indistinguishable from random. In other words, ${M_{2}}, \ldots ,{M_{n}}\mathop \equiv \limits^{{\mathrm{s}}} M{^\prime _{2}}, \ldots ,M{^\prime _{n}}{ \in _{{\mathrm{R}}}}{\{ 0,1\} ^{\rm 40\,m}}$.

It is trivial to show that ${M_{1}} \oplus \ldots \oplus {M_{n}} = 0$ through the self-inverse property of the XOR operation. In other words, ${M_{1}} = {M_{2}} \oplus \ldots \oplus {M_{n}}$. As a result, ${M_{1}}\mathop \equiv \limits^{{\mathrm{s}}} M{^\prime _{1}}$. This completes the proof. □

### Simulating a corrupted server

When $h = 1$, the server does not learn any information from the protocol besides the size of the intersection $|{X_{1}} \cap \ldots \cap {X_{n}}|$ and the size of the querier’s set $|{X_{q}}|$. We show that this is true by showing that a server with no access to the other client’s inputs can perfectly simulate the protocol’s execution.

**Theorem 1.** *There exists a simulator ${\scr S}_1$ that perfectly simulates the server’s view when $h = 1$. In other words, it holds that:*

$\eqalign{ & {\{ {\scr {S}}_1(|{X_{q}}|,|{X_{1}} \cap \ldots \cap {X_{n}}|)\} _{{X_{i}} \subseteq {\scr {U}} | i = 1, \ldots ,n}} \\ & \mathop \equiv \limits^{\rm {s} } {\{ {{\mathrm{vie}}}{{{\mathrm{w}}}_{{{\mathrm{srv}}}}}({X_{1}}, \ldots ,{X_{n}})\} _{{X_{i}} \subseteq {\scr {U}}| i = 1, \ldots ,n}}.}$

Proof. The view of the server only contains the incoming messages ${R_{i}}$ for $i = 1, \ldots ,n$ and *H*. We construct simulator ${\scr {S}}_1$ as follows:

In step 3 of the protocol, ${\scr {P}}_{{{\mathrm{srv}}}}$ receives ${R_{i}}$ for $i = 1, \ldots ,n$, which it must simulate. Knowing $|{X_{1}} \cap \ldots \cap {X_{n}}|$, the simulator randomly samples $J \leftarrow {\{ 0, \ldots ,m - 1\} ^{|{X_{1}} \cap \ldots \cap {X_{n}}|}}$, which represent the Bloom filter indices that are set to $1$. It then generates $R{ \in _{{\mathrm{R}}}}{\{ 0,1\} ^{\rm 40\,m}}$. For each $j \in J$, the simulator sets the 40-bit segment to zero: $R{[j]_{40}} \leftarrow 0 \ldots 0$. Finally, it generates ${R_{i\prime }}{ \in _{{\mathrm{R}}}}{\{ 0,1\} ^{\rm 40\,m}}$ for $i^\prime = 2, \ldots ,n$, and ${R_{1}} \leftarrow {R_{2}} \oplus \ldots \oplus {R_{n}} \oplus R$.

In step 4 of the protocol, ${\scr {P}}_{{{\mathrm{srv}}}}$ receives *H*. The simulator first sets $H \leftarrow J$, and then insert random hashes ${\scr H}_1(x){ \in _{{\mathrm{R}}}}\{ 0, \ldots ,m - 1\}$ into *H* until $|H| = |{X_{q}}|$.

We then show that these simulated incoming messages are indistinguishable from the actual messages:

From Lemma 1 we have that $\{ {M_{2}}, \ldots ,{M_{n}}\} \mathop \equiv \limits^{{\mathrm{s}}} \{ {R_{2}}, \ldots ,{R_{n}}\}$ and that ${M_{1}}\mathop \equiv \limits^{{\mathrm{s}}} {R_{2}} \oplus \ldots \oplus {R_{n}}$. If we XOR some $x$ to any ${M_{i}}$ for $i = 2, \ldots ,n$ it still holds that the result is indistinguishable from randomness. If we do the same for ${M_{1}}$, it holds that ${M_{1}} \oplus x\mathop \equiv \limits^{{\mathrm{s}}} {R_{2}} \oplus \ldots \oplus {R_{n}} \oplus x$. In other words, so long as $R\mathop \equiv \limits^{{\mathrm{s}}} {\hat Y_{1}} \cap \ldots \cap {\hat Y_{n}}$, the simulated ${R_{1}}, \ldots ,{R_{n}}$ are indistinguishable from those generated in the actual view.

Indeed, $R\mathop \equiv \limits^{{\mathrm{s}}} {\hat Y_{1}} \cap \ldots \cap {\hat Y_{n}}$ since for one hash function, the Bloom filter representing the intersection is identical to the Bloom filter that results from performing the inherent AND operation between the separately encoded Bloom filters. Since we replace the hash functions of the Bloom filter by random oracles that the server does not have access to, *J* is statistically indistinguishable from the set of actual ones in the Bloom filter.

*H* exactly corresponds to the hashes of the elements in the intersection. For the other elements $x \in X$, by replacing the hash function of the Bloom filter with a random oracle, ${\scr H}_1(x)$ are statistically indistinguishable from randomness by definition.

□

When $h > 1$, the server cannot anymore simulate the protocol’s execution without knowledge of the distribution of the elements in the parties’ sets. At this point, a protocol where clients would simply send hashes of their set elements to the server would be sufficient, and significantly cheaper when it comes to communication. On the other hand, the Bloom filter does not fully leak this information, so one might trade-off the performance gain with increasing information leakage as $h$ increases.

### Simulating corrupted clients

Finally, we show that $n - 2$ colluding clients still learn no more information than they learn from each others’ inputs. Here, we only consider the case where the querying client colludes because the other case is trivial to simulate since the other parties do not receive any incoming messages. We show that a set of at most $n - 2$ colluding clients, including the querying client, do not learn more information from the protocol than they learn from each others’ inputs, regardless of $h$.

**Theorem 2.** *There exists a simulator ${\scr {S}}_3$ that perfectly simulates the view of a set of at most $n - 2$ colluding clients, including the querying client. In other words, it holds that:*

$\eqalign{ & {\left\{ {{\scr {S}}_3({X_{i}} \ | i \in C)} \right\}_{{X_{i}} \subseteq {\scr {U}}\ | i = 1, \ldots ,n}} \\ & \mathop \equiv \limits^{\rm {s} } {\left\{ {\bigcup\limits_{i \in C} {{{\mathrm{vie}}}{{{\mathrm{w}}}_i}} ({X_{1}}, \ldots ,{X_{n}})} \right\}_{{X_{i}} \subseteq {\scr {U}} | i = 1, \ldots ,n}},}$

*for some $C \subset \{ 1, \ldots ,n\}$ with $|C| \le n - 2$ and $q \in C$*.

Proof. The view of the corrupted parties is comprised of their inputs ${X_{i}}$ for $i \in C$, the messages ${I_{x}}$ for $x \in {X_{q}}$ received by the server ${\scr {P}}_{{{\mathrm{srv}}}}$, and the output *Z*. Simulator ${\scr {S}}_3$ must therefore simulate all ${I_{x}}$, which it does as follows:

${I_{x}} \leftarrow \left\{ {\matrix{ 0 \hfill & {{\mathrm{if}}\; x\; \notin\; Z} \hfill \cr 1 \hfill & {{\mathrm{if\;}} x \in Z} \hfill \cr } } \right..$

It is trivial to see that this results in the correct output. Moreover, the simulated ${I_{x}}$ are statistically indistinguishable from the actual ${I_{x}}$; they are in fact identical, because any other ${I_{x}}$ would result in a different output. □

### Validity and practical challenges of the non-colluding server setting

The non-colluding server setting is a fundamental assumption in the proposed protocol’s security framework. First, an important distinction must be made between this non-colluding server setting and the two non-colluding server model frequently used in secure multi-party computation (MPC). The first assumes that the server does not collude with any clients, while the latter only assumes that the two servers do not collude with each other. The crucial difference is that in the typical setting for outsourced MPSI, the scheme breaks if the server colludes with anyone, while in the setting commonly used in MPC, the scheme only breaks if the server colludes specifically with the other server. As such, the latter leads to a stronger notion of security.

The non-colluding server setting achieves unlinkability efficiently by using a single cryptographic hash function unknown to the server, avoiding the high computational costs of techniques like homomorphic encryption or mixnets. However, in our protocol, *all* clients must share the same hash function, necessitating a trusted or interactive setup.

Collusion could cause a significant risk in this setting. We analyze the level of breakage if even just one client ${\scr {P}}_c$ colludes with the server. In this case, the collective secret hash functions ${\scr {H}}_j$ for $j = 1, \ldots ,h$ are leaked to the server, as well as ${X_{c}}$ and the seeds ${s_{c,i}}$ for $i = 1, \ldots ,n$. Let the server compute $R^\prime \leftarrow R \oplus {M_{c}}$, which represents the Bloom filter of the intersection of all input sets excluding that of the colluding client. From Bloom filter *R*^*′*^, the colluding parties learn $\bigcap\limits_{i \ne c} {{X_{c}}}$. Moreover, when $h > 1$, the colluding parties learn additional information about the elements contained in any of the other input sets (Vos, Conti & Erkin, 2022). For parties that collude, this means they can observe which bits are set and use that information to make inferences by comparing it with their own input sets. Because the colluding client ${\scr {P}}_c$ has access to the collective secret hash functions and the seeds used for hashing, they can reconstruct how elements are mapped and deduce with higher probability which elements belong to the other parties’ input sets. The larger $h$ is, the more bits are influenced per element, making it easier to infer missing elements through elimination. Additionally, because Bloom filters are merged using bit-wise AND operation, misaligned 1s from different input sets can accidentally indicate the presence of elements that are not actually in the intersection, leading to unintended information leakage. If a colluding client sees a certain pattern of bits in the Bloom filter, they can infer that a specific element must have originated from another client’s dataset.

In practical applications, ensuring that the server does not collude with any client poses considerable challenges. Economic or organizational incentives may drive collusion, jeopardizing the protocol’s integrity. To address these vulnerabilities, multi-server architectures that distribute trust across multiple servers under non-collusion assumptions can enhance resilience. Further improvements can be achieved by incorporating threshold models, where computations proceed only with agreement from a predefined subset of servers. This reduces the impact of a small number of colluding servers while retaining the benefits of distributed trust. Additionally, audit mechanisms, such as zero-knowledge proofs, enable clients to verify the server’s computations’ correctness without revealing additional information. However, these enhancements come at the cost of increased computational and communication complexity, which must be balanced against the protocol’s efficiency requirements.

## Results

Our open-source implementation is written in C++. We evaluate the performance of our protocol using BLAKE3 (O’Connor et al., 2021), a state-of-the-art extendable output function. We assign one thread to each client and one thread to the server. All experiments were executed on a Linux machine with Intel® Xeon® Gold 6130 CPU @ 2.10 GHz and 128 GB of memory, but only a fraction of this memory was used. Moreover, we used one core for each client and one for the server. We chose the parameters for the Bloom filters using Eq. (3). We compare the runtime performance of our protocol against Feather (Abadi et al., 2022), a recently proposed symmetric-key-based PSI protocol. Using its public implementation (https://github.com/AydinAbadi/Feather/tree/master/Feather-implementation) , we benchmark both protocols under identical conditions for varying set sizes and client counts.

### Run time without communication delays

We provide an extensive analysis of our protocol, focusing on both breakdown and total computational costs. We fix the error rate at $\varepsilon = 0.1\%$ for Bloom filters and evaluate the performance for both $h = 1$ and $h > 1$ cases. Table 3 summarizes the per-role breakdown for the server ${\scr {P}}_{{{\mathrm{srv}}}}$, the querying client ${\scr {P}}_q$, and each ${\scr {P}}_i$, highlighting the linear scalability of our protocol with respect to set sizes, numbers of clients, and hash counts. In particular, the server computation remains minimal; it stays around 3 s even in the largest configuration, which involves ${2^{16}}$ elements and ten clients. The querying client incurs the highest cost due to its central role, but the increase in computation is predictable and scales linearly with input size and hash count.

**Table 3:** Both computation (run time) and communication breakdown complexities of our MPSI for $h = 1$, $h = 5$, and $h = 10$.

			Computation (s)			Communication (MB)	
Set size	# Hash	# Client	${\scr {P}}_{{{\mathrm{srv}}}}$	${\scr {P}}_q$	${\scr {P}}_i$	${\scr {P}}_q$	${{\scr {P}}_i}$
${2^{10}}$	1	2	0.01	1.33	1.32	0.08	0.07
		3	0.02	2.01	1.83		
		4	0.02	2.10	1.74		
		5	0.03	1.80	2.01		
		10	0.05	3.13	2.76		
${2^{10}}$	5	2	0.01	1.33	1.32	0.08	0.07
		3	0.02	2.01	1.83		
		4	0.02	2.10	1.74		
		5	0.03	1.80	2.01		
		10	0.05	3.13	2.76		
${2^{10}}$	10	2	0.02	1.3	1.29	0.08	0.07
		3	0.02	1.82	1.61		
		4	0.03	1.58	1.67		
		5	0.03	1.79	1.88		
		10	0.06	2.56	2.74		
${2^{12}}$	1	2	0.05	5.16	5.16	0.3	0.28
		3	0.07	5.48	6.16		
		4	0.08	6.66	6.59		
		5	0.10	7.49	7.15		
		10	0.18	11.3	10.79		
${2^{12}}$	5	2	0.05	5.16	5.16	0.3	0.28
		3	0.07	5.48	6.16		
		4	0.08	6.66	6.59		
		5	0.10	7.49	7.15		
		10	0.18	11.3	10.79		
${2^{12}}$	10	2	0.06	5.83	5.84	0.32	0.28
		3	0.08	6.05	6.31		
		4	0.10	6.58	6.50		
		5	0.11	7.43	7.23		
		10	0.19	11.35	11.1		
${2^{14}}$	1	2	0.18	19.72	19.66	1.22	1.12
		3	0.24	25.32	24.71		
		4	0.29	26.62	25.80		
		5	0.37	28.24	28.08		
		10	0.70	45.74	43.56		
${2^{14}}$	5	2	0.18	19.72	19.66	1.22	1.12
		3	0.24	25.32	24.71		
		4	0.29	26.62	25.80		
		5	0.37	28.24	28.08		
		10	0.70	45.74	43.56		
${2^{14}}$	10	2	0.21	20.57	20.42	1.3	1.12
		3	0.28	23.98	22.87		
		4	0.34	26.64	25.85		
		5	0.41	25.50	28.39		
		10	0.73	39.6	38.53		
${2^{16}}$	1	2	0.75	76.04	76.92	4.87	4.49
		3	1.05	90.39	98.27		
		4	1.30	106.09	105.88		
		5	1.68	114.77	113.51		
		10	3.08	188.3	173.74		
${2^{16}}$	5	2	0.75	76.04	76.92	4.87	4.49
		3	1.05	90.39	98.27		
		4	1.30	106.09	105.88		
		5	1.68	114.77	113.51		
		10	3.08	188.3	173.74		
${2^{16}}$	10	2	0.92	82.36	82.08	5.18	4.49
		3	1.22	100.76	94.87		
		4	1.52	105.58	102.72		
		5	1.79	118.76	113.93		
		10	3.25	175.52	173.67		

Table 4 presents the total execution times in various settings. For example, when ten clients each hold ${2^{14}}$ elements and the hash count is $h = 10$, the total computation time remains under 54 s. As expected, increasing set sizes impact all parties, especially the clients and querying client, whose runtime grows proportionally with the input. Similarly, more clients introduce additional overhead, though the impact remains smooth and predictable. This demonstrates that even in relatively large-scale scenarios, our protocol remains practical and efficient for real-world deployment.

**Table 4:** Total run time for $h = 1$, $h = 5$, and $h = 10$ when communication is instant, averaged over 5 experiments. An intersection with ten clients, each with more than 16,000 elements, requires only 54 s to compute.

Set size	# Client	Run Time (s)		
		$h = 1$	$h = 5$	$h = 10$
${2^{10}}$	2	1.74	1.42	1.39
	3	2.13	2.12	1.94
	4	2.43	2.25	1.86
	5	2.42	2.53	2.33
	10	3.58	3.68	3.54
${2^{12}}$	2	6.61	5.55	6.23
	3	6.94	6.98	7.18
	4	7.27	7.78	7.48
	5	9.12	8.93	8.92
	10	13.91	13.28	13.61
${2^{14}}$	2	25.20	21.23	22.08
	3	26.81	28.80	27.30
	4	30.48	29.69	29.86
	5	35.85	34.09	34.16
	10	53.73	53.46	53.08
${2^{16}}$	2	94.60	82.76	88.46
	3	120.54	110.07	112.12
	4	128.28	128.86	115.24
	5	144.14	172.14	140.28
	10	212.38	215.42	212.72

**Figure 2:**
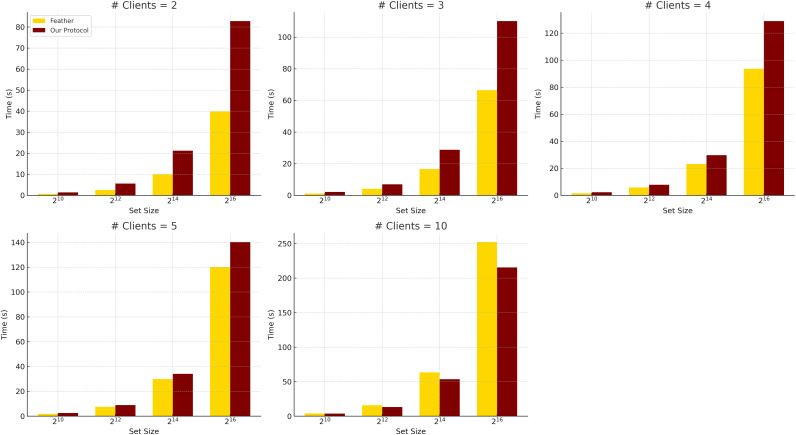
Total run time comparison between Feather and our MPSI when communication is instant, averaged over five experiments (h = 10).

*Comparison with Feather*. Feather performs well for two-client settings with small input sizes, completing a ${2^{10}}$-sized PSI in less than 1 s, as shown in Table 5. Figure 2 illustrates this scalability difference, comparing the total runtime of Feather and our protocol across varying clients and set sizes. However, it scales poorly when increasing the number of clients due to its pairwise intersection model and reliance on modular arithmetic and permutations.

**Table 5:** Feather protocol performance results (in s).

Set size	# Client	Run Time (s)	Set size	# Client	Run Time (s)
${2^{10}}$	2	0.74	${2^{14}}$	2	9.89
	3	1.06		3	16.73
	4	1.47		4	23.25
	5	1.87		5	29.74
	10	3.91		10	62.76
${2^{12}}$	2	2.51	${2^{16}}$	2	40.15
	3	4.14		3	65.81
	4	5.92		4	93.38
	5	7.47		5	120.10
	10	15.57		10	253.58

Using lightweight XOFs and XOR operations, our protocol shows better scalability and comparable performance in many settings. In addition, our protocol exhibits superior scalability concerning the number of clients. For instance, at $k = {2^{16}}$ and $n = 10$, our protocol completes in 212.72 s, compared to Feather’s 253.58 s. While the advantage is modest at this point, the performance gap continues to widen as $n$ increases, thanks to our aggregated masking approach, which avoids redundant computations and communications. Notice that while Feather has strong performance for $n = 2$, our protocol offers more consistent scaling across all values of $n$ and $k$, maintaining competitive performance without sacrificing flexibility or simplicity.

We note that the Feather results may not exactly match those in its original publication, as we used different hardware and the original evaluation parameters were not fully disclosed.

### Concrete communication cost analysis

Table 3 presents the per-client communication costs, distinguishing between the querying client ${\scr {P}}_q$ and the regular clients ${\scr {P}}_i$. In our protocol, the server ${\scr {P}}_{{{\mathrm{srv}}}}$ sends a single message to ${\scr {P}}_q$, which is included in ${\scr {P}}_q$’s total communication. The cost for regular clients remains constant for a given set size and number of hash functions, whereas ${\scr {P}}_q$’s cost increases linearly with the number of hash functions due to element replication. For example, in the largest tested configuration ( $k = {2^{16}}$, $h = 10$), ${\scr {P}}_q$ transmits approximately 5.18 MB, while each regular client transmits approximately 4.49 MB. This per-client overhead is independent of the total number of participants. However, as shown in Table 6, the overall communication increases linearly with the number of clients, demonstrating the protocol’s scalability and efficiency in bandwidth-constrained, multi-party scenarios.

**Table 6:** Total communication cost comparison (in MB) between Feather and our MPSI for $ h\bf = 10$.

Set size	# Client	Our MPSI (MB)	Feather (MB)
${2^{10}}$	2	0.14	0.2
	3	0.21	0.2
	4	0.28	0.2
	5	0.35	0.2
	10	0.7	0.2
${2^{12}}$	2	0.56	1.4
	3	0.84	1.4
	4	1.12	1.4
	5	1.4	1.4
	10	2.81	1.4
${2^{14}}$	2	2.25	5.8
	3	3.37	6.0
	4	4.49	6.0
	5	5.62	6.0
	10	11.23	6.2
${2^{16}}$	2	8.99	23.8
	3	13.48	24.0
	4	17.97	24.0
	5	22.47	24.2
	10	44.93	25.0

**Figure 3:**
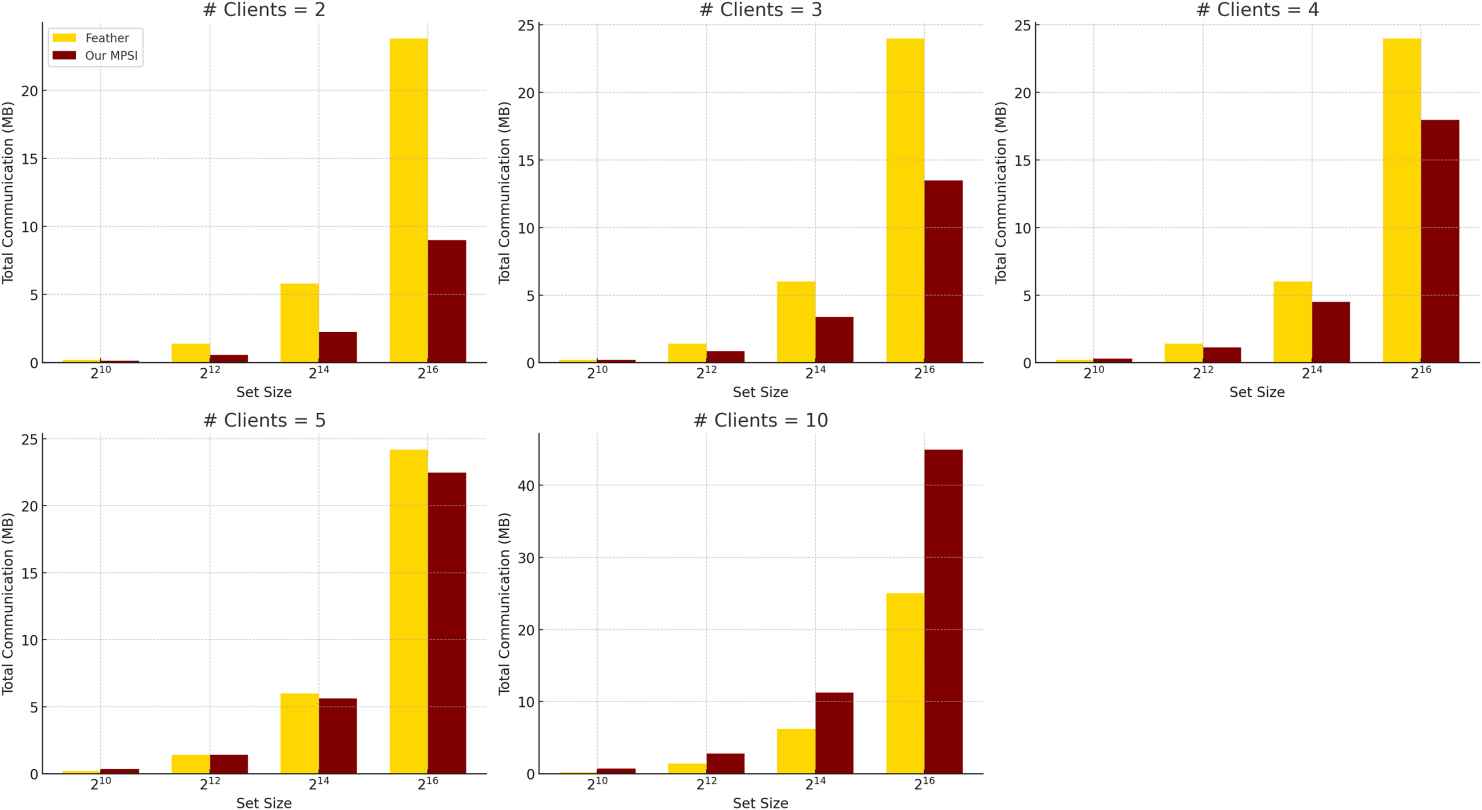
Total communication complexity comparison between feather and our protocol when communication is instant, averaged over five experiments ( ${ h}\bf = 1$).

*Comparison with Feather*. We further compare the communication efficiency of our protocol with Feather (Abadi et al., 2022), as summarized in Table 6 and illustrated in Fig. 3, using identical evaluation settings. Feather achieves low and stable communication overhead for small set sizes, but its cost increases sharply with larger datasets, even with a fixed number of clients. In contrast, our protocol incurs slightly higher per-client communication but scales linearly and predictably with both the number of clients and the set size. This makes it more suitable for large-scale deployments where bandwidth predictability and scalability are critical.

As shown in Table 3, increasing the number of hash functions $h$ does not consistently improve computational performance. In some smaller configurations, higher $h$ values show marginal improvements. However, for larger set sizes, computation time tends to increase or plateau. These observations suggest that while increasing $h$ may reduce false positives, it does not provide meaningful performance gains and may even introduce additional computational overhead in practice.

### Run time with simulated communication delays

**Figure 4:**
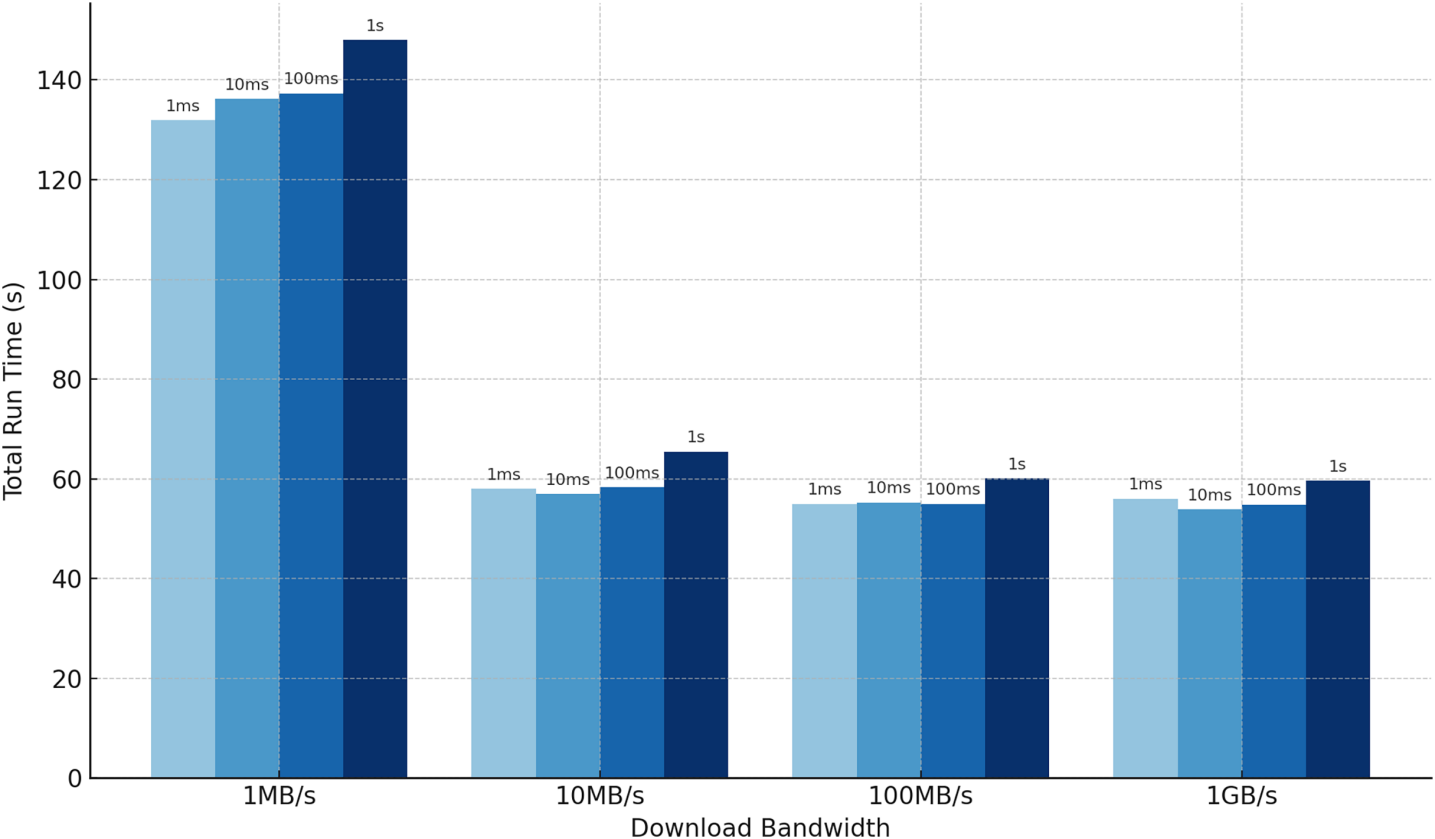
Our total run time when communication incurs latency and bandwidth delays, averaged over five experiments. The numbers above the bars indicate the latency. An intersection with 10 clients, each holding 16,384 elements requires less than 150 s to compute when bandwidth is limited to 1 MB/s and latency is 1 s.

One often-overlooked aspect of outsourced private set intersection protocols is the impact of communication delays. To address this, we analyze the runtime of our protocol under varying download bandwidth and latency constraints, which are uniformly applied across all communication channels in the star topology. We conduct experiments with $n = 10$ clients and a set size of $k = {2^{14}}$, fixing the Bloom filter error rate at $\varepsilon = 0.1\%$ and focusing on cases where $h > 1$. As shown in Fig. 4, bandwidth limitations significantly affect efficiency. For $h = 10$, the resulting Bloom filter size is approximately $m = 235,568$ bins, meaning each client sends about $\rm 40 \,m$ bits, or roughly 1.12 MB (see Table 3). Since we simulate download bandwidth, the server must process incoming data sequentially, introducing delays. In contrast, latency has minimal effect, as the protocol requires only a single round-trip interaction and thus incurs latency just twice. Detailed numerical results of these latency and bandwidth experiments are provided in Table A1 in the Appendix.

### Performance evaluation of BLAKE3-XOF

**Figure 5:**
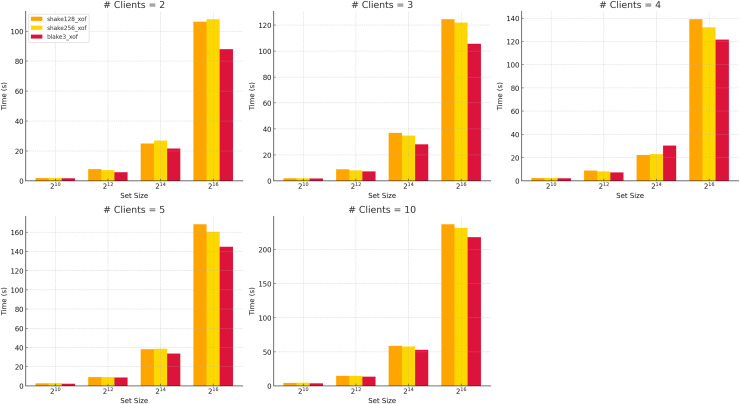
Run time of different XOFs *vs* number of clients for selected dataset sizes.

All analyses in this study use the BLAKE3-XOF (O’Connor et al., 2021) function, which offers a modern cryptographic design characterized by strong security guarantees and a high potential for parallelization. As illustrated in Fig. 5, comparative runtime performance measurements reveal that BLAKE3-XOF consistently outperforms both SHAKE128-XOF and SHAKE256-XOF (National Institute of Standards and Technology (NIST), 2015) (see also Table A2) across a wide range of data set sizes and client numbers. While SHAKE-based XOFs offer strong cryptographic security and are well-established, the performance advantage of BLAKE3-XOF is evident even at small dataset sizes and low party counts. It becomes increasingly pronounced as both parameters grow. This suggests excellent scalability and low computational overhead, making it a highly efficient choice for large-scale or concurrent applications.

Although integrating BLAKE3’s SIMD-friendly design could have accelerated the protocol, we used the same setup of hash functions in Feather to ensure a fair comparison. Future work may explore BLAKE3-specific optimizations.

## Conclusion

Private set intersections are a crucial functionality in everyday applications, yet executing these protocols remains costly. In this work, we demonstrate that with a trusted, non-colluding server, the problem becomes far more efficient to evaluate. However, we also highlight the challenges associated with this assumption. Specifically, the unlinkability property—ensuring the server learns nothing about the set elements—only holds if no client colludes with the server. We show that under this assumption, our protocol can intersect five datasets with 16,000 elements in just 35 s. Moreover, if minor information leakage regarding the distribution of elements among parties is tolerated, the runtime can be further optimized. Lastly, we have open-sourced our proof-of-concept implementation to facilitate future research and advancements in this area. In future work, we aim to explore multi-server architectures to mitigate collusion risks and investigate techniques to reduce or eliminate the leakage of intersection size and access patterns without sacrificing performance. Additionally, we plan to explore parallel implementations using BLAKE3-XOF to further improve run time scalability.

## Appendix: detailed performance tables

**Table A1:** Run time (in s) under varying network conditions for set size ${2^{14}}$ with 10 clients and BLAKE3-XOF (h = 10).

Bandwidth (MB/s)	Latency (ms)	Run Time (s)	Bandwidth (MB/s)	Latency (ms)	Run Time (s)
1	1	131.92	100	1	54.96
	10	136.24		10	55.28
	100	137.32		100	54.98
	1,000	148.01		1,000	60.04
10	1	57.98	1,000	1	56.01
	10	56.94		10	53.85
	100	58.30		100	54.82
	1,000	65.44		1,000	59.61

**Table A2:** Comparison of the run time (in s) of our MPSI for different XOFs across various set sizes and party counts.

Set size	# Client	BLAKE3-XOF	SHAKE128-XOF	SHAKE256-XOF
${2^{10}}$	2	1.73	1.90	1.80
	3	1.82	1.98	1.91
	4	2.19	2.39	2.39
	5	2.33	2.59	2.79
	10	3.82	4.45	4.65
${2^{12}}$	2	5.82	7.82	7.12
	3	7.24	8.92	8.12
	4	7.32	8.92	8.12
	5	8.85	9.26	9.20
	10	13.70	14.82	14.66
${2^{14}}$	2	21.66	24.93	26.85
	3	28.11	36.95	34.83
	4	30.43	22.32	22.94
	5	33.64	38.25	38.60
	10	52.80	58.71	57.74
${2^{16}}$	2	87.99	106.36	108.08
	3	105.55	124.58	121.98
	4	121.60	139.19	132.06
	5	144.94	168.37	160.36
	10	218.09	237.25	232.00

## Additional Information and Declarations

### Data Availability

The following information was supplied regarding data availability:

Raw data and code is available at GitHub: https://github.com/aslibay/Outsourced-MPSI-May25.
